# Low-Dose CT Image Post-Processing Based on Learn-Type Sparse Transform

**DOI:** 10.3390/s22082883

**Published:** 2022-04-09

**Authors:** Wenfeng Zheng, Bo Yang, Ye Xiao, Jiawei Tian, Shan Liu, Lirong Yin

**Affiliations:** 1 School of Automation, University of Electronic Science and Technology of China, Chengdu 610054, China; winfirms@uestc.edu.cn (W.Z.); yexiao@std.uestc.edu.cn (Y.X.); jravis.tian23@gmail.com (J.T.); shanliu@uestc.edu.cn (S.L.); 2 Department of Geography and Anthropology, Louisiana State University, Baton Rouge, LA 70803, USA

**Keywords:** low dose CT, sparse representation, sparse transform, image decomposition theory

## Abstract

As a detection method, X-ray Computed Tomography (CT) technology has the advantages of clear imaging, short detection time, and low detection cost. This makes it more widely used in clinical disease screening, detection, and disease tracking. This study exploits the ability of sparse representation to learn sparse transformations of information and combines it with image decomposition theory. The structural information of low-dose CT images is separated from noise and artifact information, and the sparse expression of sparse transformation is used to improve the imaging effect. In this paper, two different learned sparse transformations are used. The first covers more organizational information about the scanned object. The other can cover more noise artifacts. Both methods can improve the ability to learn sparse transformations to express various image information. Experimental results show that the algorithm is effective.

## 1. Introduction

The CT image technology has unique advantages and practical value [1,2,3,4]. CT image has multi-level attributes compared with X-ray detection, and CT has high-density resolution and sensitivity. It can clearly show the tissue characteristics and pathological changes of some organs composed of soft tissue [5,6,7]. Because of its high resolution and sensitivity, CT examination is superior to X-ray imaging examination. X-ray is more used for bone examination, while CT examination covers almost all human body parts, such as the brain, chest, blood vessels, and nervous system [8,9,10]. These advantages make CT imaging technology occupy an irreplaceably important position in clinical examination, so more and more disease diagnoses and treatments need CT examination for assistance.

There are three methods to improve the image quality of low-dose CT images obtained in the low tube current situation, i.e., the restoration of projection data, iterative reconstruction algorithm, and low-dose CT image post-processing [11,12,13,14,15,16]. The low-dose CT image post-processing method takes the reconstructed low-dose CT image as the original image. It takes an appropriate image processing algorithm to remove noise and artifacts, improve image quality, and restore tissue information [17,18,19,20]. The purpose is to make the image quality after processing as close as possible to the image quality of standard-dose CT. Because there is no need to obtain the original projection data, this method can obtain the experimental data more easily than the previous two methods without CT equipment or original projection data. Moreover, the statistical iterative reconstruction algorithm needs more storage space and more computing time in practical application. Simultaneously, the image post-processing method can be offline processing with a higher application value. Decreasing tube current or tube voltage during CT scans causes photon number loss that adds additional noise to the projection data. The statistical law of noise basically conforms to Gaussian distribution and composite Poisson distribution. However, in the process of image reconstruction, after continuous projection and back-projection transformation, the noise mainly presents in the form of strip artifacts and unsteady speckle noise in low-dose CT images. These noises and artifacts are difficult to remove under the premise of retaining tissue details and edges.

In recent years, many algorithms and models have been used to restore low-dose CT images in the field of sparse representation. Among them, the two most famous models are the synthesis model and analysis model [21], also called Analytical Dictionary [22,23,24,25], and synthesis dictionary in some works of literature. Because of the above model, many processing algorithms related to low-dose CT images are proposed. For example, Xu et al. [26] proposed a penalty-weighted least squares method. This method takes the super complete composite dictionary as the regularization term. It applies dictionary learning to 2D low-dose CT image reconstruction. The dictionary learning training set can be obtained from the CT images reconstructed by filtering the back-projection algorithm in the standard dose scene. The global dictionary can be trained by extracting two-dimensional image blocks or an adaptive dictionary jointly estimated with low-dose CT images. The global dictionary is better than the adaptive estimation dictionary in the low-dose scanning scenario. Furthermore, several studies have proposed 3D CT reconstruction by learning 3D dictionaries from 3D image blocks or 3D/2D dictionaries extracted from slices along the X-Y, the Y-Z, and the X-Z directions [27]. However, the post-processing of low-dose CT images based on the above two models cannot guarantee the convergence of constraints. Therefore, Zheng et al. proposed a low-dose CT image reconstruction algorithm based on learning sparse transform to solve the convergence problem of the regularization term. This algorithm proved the effectiveness of low-dose CT image processing based on learning sparse transformation [28].

This paper’s main research content is the degradation of CT image quality in the scene of low-dose CT scanning, which reduces tube current. The decrease of image quality is mainly caused by the interference of noise and artifact caused by photon number reduction, which affects clinical pathology diagnosis and analysis. A new image post-processing method based on learning sparse transformation is proposed in this paper to solve the above problem. This method combines the knowledge of sparse representation with the image morphological component analysis method. The structure sparse and sparse noise transform is constructed to represent the CT image differently to separate the information and remove the noise and artifact. The experimental results show that the proposed algorithm can effectively improve the quality of low-dose CT images and perform better in the evaluation index.

## 2. Materials and Methods

### 2.1. Datesets

#### 2.1.1. Equipment Introduction

There are three processes for CT equipment to generate images: data acquisition, image reconstruction, and image display. The CT simulation image acquisition method used in this paper can refer to [29].

Data acquisition refers to the process by which an X-ray with initial intensity is emitted from an X-ray source and passes through the scanning object (such as the human body). The detector receives the X-ray with intensity attenuation. The specific attenuation degree is related to the nature of the scanned object and the path it passes. The radiation source and the detector rotate at equal intervals during this period. The detector collects data after scanning a circle around the object. Image reconstruction solves the attenuation rate of X-ray after scanning the object. Because most tissues of the human body are very close to that of water, most tissues’ attenuation coefficient increases the difference between tissues. The concept of CT value is introduced. The unit is HU (Hounsfield). $H=1000\left(\left(\mu_{-}t-\mu_{-}W\right)\right)/\mu \_W$, where $\mu_{-}t$ represents the attenuation rate of tissue, and μ_W represents the attenuation rate of water.

**Figure 1:**
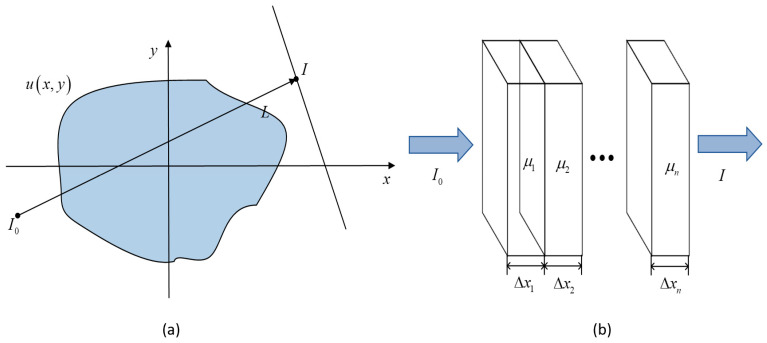
Schematic diagram of X-ray penetration of heterogeneous materials. (**a**) for non-uniform plane, (**b**) for discrete organization.

The mathematical expression of the CT image reconstruction process can be expressed according to Lambert–Beer law [30]. Assuming that the object through which the X-ray passes is a homogeneous material, as shown in Figure 1, the continuous representation of the attenuation path L of the X-ray is as follows:

(1) $$I=I_{0}e^{-\int_{L}\mu \left(X,y\right)dL}=I_{0}e^{-y_{L}}$$

In the formula, the initial energy of the X-ray is $I_{0}$, the X-ray energy received by the detector at a rotation angle, that is I, μ(X,y) is the attenuation rate of tissues inside the scanning object, $\int_{L}\mu \left(X,y\right)dl$ is called radon transformation. Transformation is also known as a projection of μ(X,y). Since computers operate in the form of a matrix, it is necessary to discretize it, $\mu_{i}$ represents the attenuation coefficient after discretization, and $\Delta X_{i}$ represents the X-ray penetration path after discretization. For example, the representation after discretization is as follows:

(2) $$I=I_{0}e^{-\sum_{i=1}^{n}\mu_{i}\Delta X_{i}}=I_{0}e^{-y_{L}}$$

#### 2.1.2. Low Dose CT Image Simulation

When studying low-dose CT images, we often need to use the experimental data of standard-dose CT and low-dose CT of the same individual for algorithm research or image quality evaluation. However, in practice, if the subject is subjected to two consecutive normal-dose CT examinations and low-dose CT examinations, the subject will receive excessive X-ray radiation and increase the risk of cancer. Therefore, the low-dose CT images used in this paper were generated by simulation.

There are many ways to simulate low-dose CT. The first method utilizes the GE Noise Addition Tool from GE Healthcare, Waukesha, WI, USA) to simulate low-dose CT images. This software tool generates sinograms from standard dose CT images and estimates additional noise in the sinograms based on the reduced exposure dose. Although very simple, this method is only applicable to CT images obtained by GE scanners, and is also limited to specific suppliers [31].

The second method is to process the projection data when performing conventional-dose CT scans. This method obtains its projection data directly from the CT equipment and adds noise information to simulate the low-dose CT scan projection data. Although this method can restore the most realistic low-dose CT situation, it is very difficult to obtain projection data from CT equipment. This limitation raises the research threshold of low-dose CT and is not conducive to the development of this field [13,32].

Finally, Won Kim C. et al., proposed a method that does not require the original projection data, aiming at the difficulty of obtaining projection data for CT equipment. This method measures the noise equivalent quanta (NEQ) and modulation transfer function (MTF) of CT systems under different combinations of target attenuation and tube current by analyzing the noise power spectrum (NPS) of CT images obtained with a set of phantoms. The noise equivalent quantum is positively correlated with the noise equivalent quantum, which is characterized by representing the noise equivalent density of each CT image. The modulation transfer function is negatively correlated with the dose, reflecting the texture characteristics of the noise. These measurements were used to build a comprehensive CT noise model including reduced X-ray photon flux, object attenuation, system noise, and bowtie filters. They then used synthetic noise maps generated from reference CT images to generate simulated noise signal maps for dose reduction conditions [33].

The whole simulation process is divided into two stages. The first step is to obtain the parameters in the noise model. After the noise parameters are extracted by performing multiple CT scans on a set of models, this paper calculates the noise power spectrum of the acquired CT image data set, and estimates the NEQ and MTF of a set of noise based on the noise power spectrum data set. Finally, the curve fitting technique is used to calculate the noise equivalent quantum to finally determine various parameters in the noise model.

### 2.2. Methods

In the 1990s, Olshausen and Field [22] published a paper that explained the image signal’s sparse expression from the biological point of view. We call the set of essential functions a dictionary, so the sparse transformation of the signal is divided into two parts: the dictionary’s construction the representation of sparse coding. According to the different structures of dictionaries, they can be divided into analytic dictionaries and composite dictionaries. However, compared with the lack of self-adaptability of the Analytical Dictionary and the slow convergence speed of the synthesis dictionary, the learning sparsity applied in this paper has a larger adaptive range and faster iteration speed.

#### 2.2.1. Low Dose CT Image Processing Based on Learning Sparse Transform

Assuming that the original image is a matrix X of N×T, the sparse transformation is a M×N dimensional matrix W. The image after sparse transformation M×T (sparse coding) is Z and column sparse, the error of sparse representation is expressed as $\underset{W,Z}{min}\|WX-Z{\|}_{F}^{2}$, where $\|\cdot {\|}_{F}$ is the Frobenius norm. When the sparse transformation matrix is unknown, model the image signal sparse transformation solution is as follows

(3) $$\underset{W,Z}{min}\|WX-Z{\|}_{F}^{2}\;s.t.\|Z_{t}{\|}_{0}\leq s\;\forall t$$

where $Z_{t}$ is the t column of the Z matrix. The above formula can be interpreted as the natural image or signal representing less non-zero elements in a certain transformation domain. The remaining elements represent the edge error information of the image. Td the sparse representation of the image signal X can be realized by continuously adjusting W and Z. However, Equation (1) has a set of trivial solutions. That is, when W and Z are all zero matrices, the constraint can also be satisfied. Adding a penalty term to the sparse transformation solving model is necessary. In the classical synthesis dictionary model, we can avoid the appearance of the trivial solution by the norm constraint of sparse transformation W. However, this method cannot avoid non-full rank [34], so we need to add a full rank constraint. The full rank constraint is a non-convex problem, which is not easy to solve. Therefore, the sparse transformation matrix in Equation (1) is assumed to be a square matrix for convenience. When W is a square matrix, (M=N), the equivalent full rank constraint of W can be realized by the negative logarithmic determinant constraint [35]. Therefore, the sparse transformation of the image in Equation (1) can be further modeled as follows:

(4) $$\underset{W,Z}{min}\|WX-Z{\|}_{F}^{2}-\lambda logdet\left(W\right)+\mu \|W{\|}_{F}^{2}\;s.t.\|Z_{t}{\|}_{0}\leq s\;\forall$$

where λ and μ are the penalty coefficients, respectively. According to machine learning theory [36], the non-convex norm constraint $L_{0}$ of the above formula can be replaced by the norm constraint $L_{1}$ to facilitate the solution.

(5) $$\underset{W,Z}{min}\|WX-Z{\|}_{F}^{2}-\lambda logdet\left(W\right)+\mu \|W{\|}_{F}^{2}+\eta \overset{T}{\underset{t=1}{\sum }}\|Z_{t}{\|}_{1}$$

where η is the sparse penalty term corresponding to 1 norm constraint. The purpose of the model is to minimize the sparse error, control the sparsity degree of the matrix after sparse transformation, and eliminate the problem that the sparse transformation matrix is not full of rank.

#### 2.2.2. Image Processing Algorithm Based on Learning Sparse Transform

The above learning sparse transform methods can be used for image denoising. The noise of low-dose CT images consists of non-directional speckles. The artifact is strong, the amplitude range is large, and the directionality is obvious. The direct application of the above method has limited denoising effect. According to the image morphological component analysis [37,38,39,40], a low-dose CT image can be equivalent to the sum of tissue components and noise artifacts. There is a specific basis function for different components that can sparse represent the component.

In contrast, the basis function cannot represent other components; that is, their corresponding basis functions can only represent different components. Therefore, different components in low-dose CT images can be distinguished by constructing different basis functions. Therefore, in this section, the sparse transformation matrix corresponding to the tissue component and the noise component is trained respectively to realize the decomposition of image components and achieve a better denoising effect of low-dose CT images.

According to the idea of image morphological component analysis [41], low-dose CT images $X_{ld}$ can be divided into two parts: the image of tissue structure information $X_{hd}$ and the image of noise artifact information $X_{na}$. Therefore, low-dose CT images can be represented as follows:

(6) $$X_{ld}=X_{hd}+X_{na}$$

The result of CT image processing in this section is to recover the tissue structure information image in low-dose CT image $X_{hd}$.

At this time, the low-dose CT image post-processing algorithm model based on learning sparse transform is as follows:

(7) $$\underset{X_{ld,t}}{min}\lambda \|X_{hd}+X_{na}-X_{ld}{\|}_{2}^{2}+\|W_{hd}P_{j}X_{hd}-Z_{t}^{hd}{\|}_{2}^{2}+\|W_{na}P_{j}X_{na}-Z_{t}^{na}{\|}_{2}^{2}\;s.t.\|Z_{t}{\|}_{0}\leq s\;\forall t$$

Among them, $Z_{t}=\left[Z_{t}^{hd},Z_{t}^{na}\right]$. $W_{hd}$ and $W_{na}$ represent the standard dose CT image and noise information image sparse transformation matrix, respectively. The specific solution method is the optimal gradient descent method from the standard CT image and CT noise image sets. For the N×N operator of extracting image block $P_{j}$, the image is cut into image blocks for processing, realizing parallel processing. $Z_{t}^{hd}$ and $Z_{t}^{na}$ represent the sparse representation coefficient of tissue structure $X_{ld}$ and noise artifact, respectively.

Step 1: the formula of the sparse coefficient of organizational structure information $Z_{t}^{hd}$ is as follows:

(8) $$Z_{t}^{hd}=\{\begin{array}{c} W_{hd}P_{j}X_{ld},{\left|W_{hd}P_{j}X_{ld}\right|}_{2}\geq \eta \\ 0,\;{\left|W_{hd}P_{j}X_{ld}\right|}_{2}<\eta \end{array}$$

Step 2: the solution formula of noise artifact sparse coefficient $Z_{t}^{na}$ is as follows:

(9) $$Z_{t}^{na}=\{\begin{array}{c} W_{na}P_{j}X_{ld},{\left|W_{na}P_{j}X_{ld}\right|}_{2}\geq \eta \\ 0,\;{\left|W_{na}P_{j}X_{ld}\right|}_{2}<\eta \end{array}$$

Step 3, organize the image of structure information $X_{hd}$ and noise artifact information $X_{na}$ to obtain the derivation of (5)

(10) $$\left[\begin{array}{c} X_{hd}\\ X_{na} \end{array}\right]=H^{-1}\left[\begin{array}{c} \lambda X_{ld}+\overset{K}{\underset{i=1}{\sum }}{\left(W_{hd}P_{j}\right)}^{T}Z_{t}^{hd}\\ \lambda X_{ld}+\overset{K}{\underset{i=1}{\sum }}{\left(W_{na}P_{j}\right)}^{T}Z_{t}^{na} \end{array}\right]$$

(11) $$H=\left[\begin{array}{cc} \lambda I+\overset{K}{\underset{i=1}{\sum }}{\left(W_{hd}P_{j}\right)}^{T}W_{hd}P_{j} & \lambda I\\ \lambda I & \lambda I+\overset{K}{\underset{i=1}{\sum }}{\left(W_{na}P_{j}\right)}^{T}W_{na}P_{j} \end{array}\right]$$

**Figure 2:**
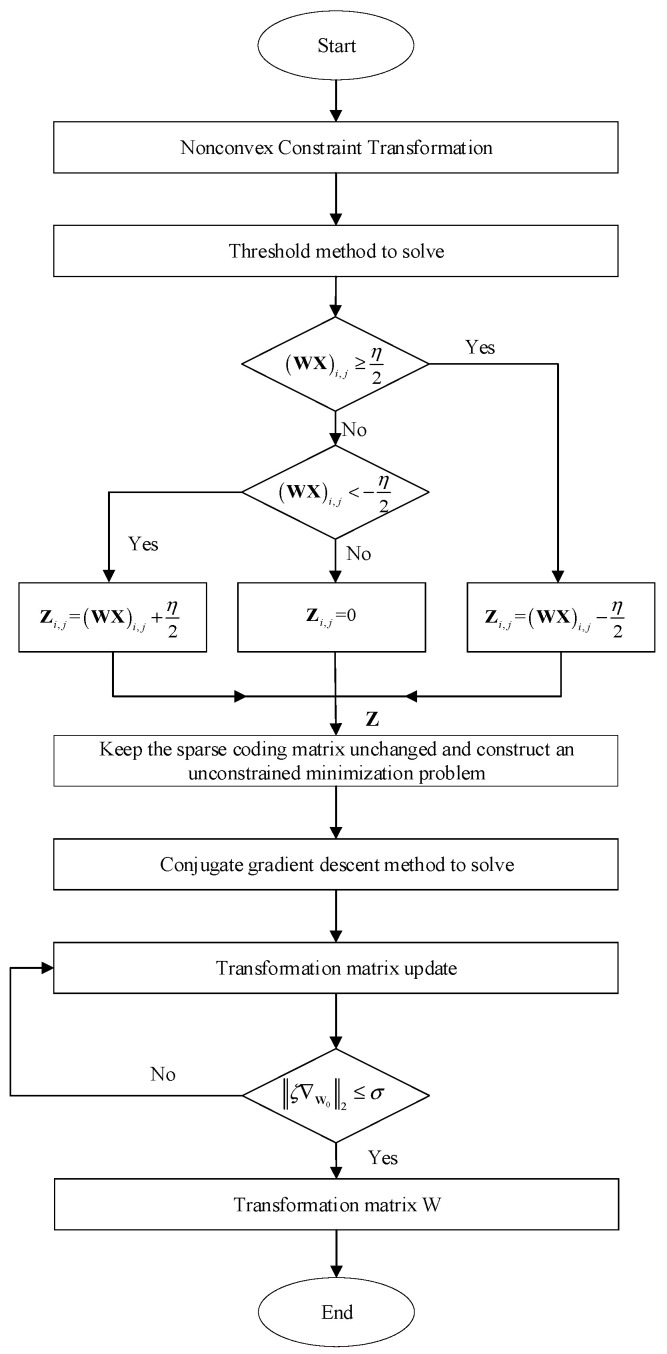
Flowchart of solving sparse transformation.

The solution process of sparse transformation matrix ***W*** and sparse coefficient ***Z*** is shown in Figure 2.

### 2.3. The Construction Method of Learning Sparse Transformation

The performance of the discriminative learning sparse transformation model is closely related to the discernible sparse transformation matrix. The training data for learning the discriminative sparse transform is from the fan beam-scanning heart trunk phantom CT images that were reconstructed by filtered back projection. The noise characteristics of different CT equipment are different, so the sample data and experimental data must come from the same CT equipment. In order to make the tissue structure sparse transformation matrix $W_{hd}$ and the sparse noise transformation matrix $W_{na}$ better express the tissue information and noise information, the standard-dose CT image and the noise CT image generated by the difference between the standard-dose CT image and the simulated low-dose CT image are used to train the sparse transformation matrix.

1. The standard-dose projection data was obtained by simulating the fan beam scanning with the CT image of the sample standard dose;
2. Based on the NEQ and MTF characteristics of CT equipment, a Gaussian noise model is constructed to simulate the low dose projection data;
3. The filtered back-projection algorithm is used to reconstruct the low-dose CT image of the sample from the low-dose CT projection data;
4. The CT noise image is obtained by subtracting the sample standard-dose CT image and the sample low dose CT image.

**Figure 3:**
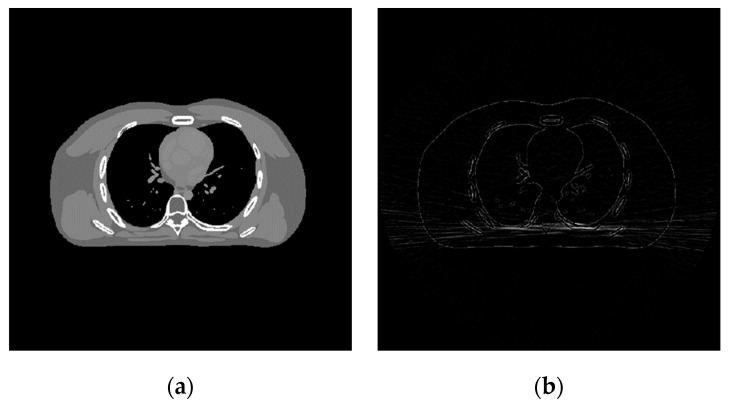
Training image. (**a**) Standard dose; (**b**) noise CT images.

After the sample data construction is completed, the standard dose CT images are processed to reduce the calculation cost, as shown in Figure 3. Next, noise CT images were extracted according to the image block’s specific size. Finally, the alternating algorithm for sample image blocks obtains the discriminative sparse transformation matrix.

## 3. Experiments and Results

### 3.1. Dataset and Experimental Environment

This paper’s test data are low-dose CT images obtained by simulating the heart trunk phantom CT data’s fan-beam scanning scene [42,43,44]. This study used simulated images, so it may not be as effective as expected in a clinical setting. The image is different from the sample image compare to the classical CT reconstruction algorithm filtering back projection (Hanning window), image post-processing algorithm based on (Discrete Cosine Transform) DCT [45], and penalty weighted least square reconstruction method based on edge-preserving regularization (EP), where EP expression is $R(x)=\sum_{j=1}^{N_{P}}\sum_{k\in N_{j}}\kappa_{j}\kappa_{k}\varphi \left(x_{j}-x_{k}\right)$. It is used to control the neighborhood range. $\kappa_{j}$ and $\kappa_{k}$ are the parameter of excitation noise equalization, and $\varphi \left(t\right)≜\delta^{2}\left(\left|\frac{t}{\delta }\left|-log(1+\right|\frac{t}{\delta }\right|\right))$.

The experimental hardware environment is core i5, 3.7GHz, six-core CPU, 16GB memory. The software environment is the Linux system and MatLab programming platform.

### 3.2. Visual Effect Analysis of CT Image

**Figure 4:**
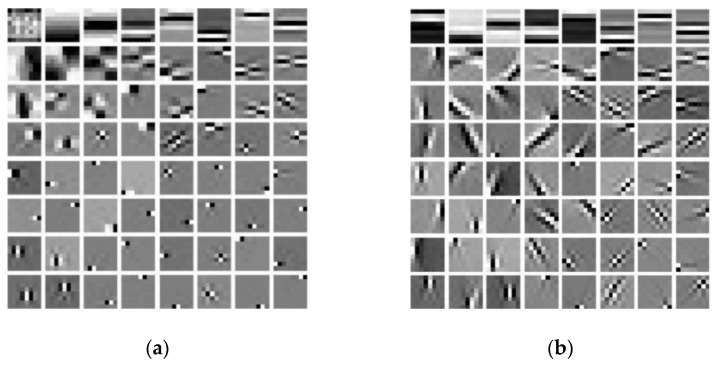
Shows the sparse transformation. (**a**) Structure sparse; (**b**) Noise sparse.

During the discriminative sparse transformation training, the sample data are also 5 CT images of the heart torso phantom. Three of them are used for training and two are used for testing. The image blocks are extracted according to the size and step size of 1. In the process of solving the alternating iteration algorithm, the number of iterations is set to 1000, the sparse threshold η is set to 126, and the sum of penalty coefficients λ is equal μ as $5.85\times 10^{15}$. As shown in Figure 4, the results of sparse structural transformation and noise sparse transformation are displayed. The size of each transformed image block is 8×8.

**Figure 5:**
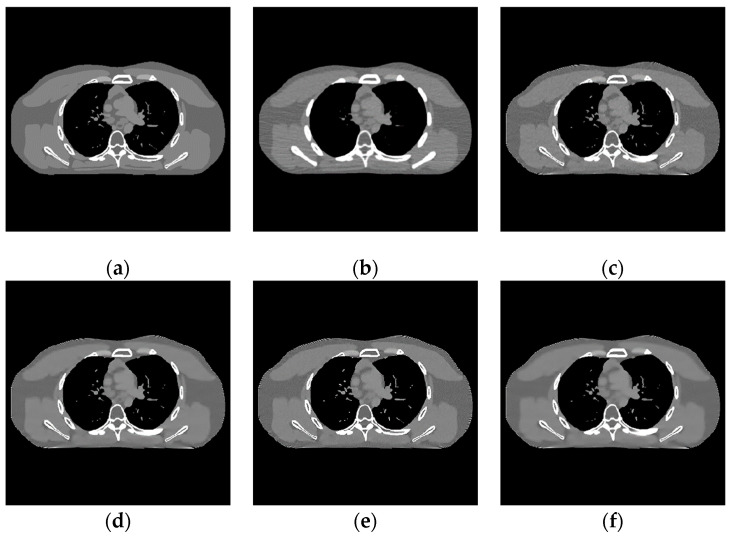
Experimental results of incident photon number $10^{5}$. (**a**) real image; (**b**) FBP reconstruction image; (**c**) EP iterative reconstruction image; (**d**) DCT image post-processing; (**e**) DL iterative reconstruction; (**f**) learning sparse transform.

**Figure 6:**
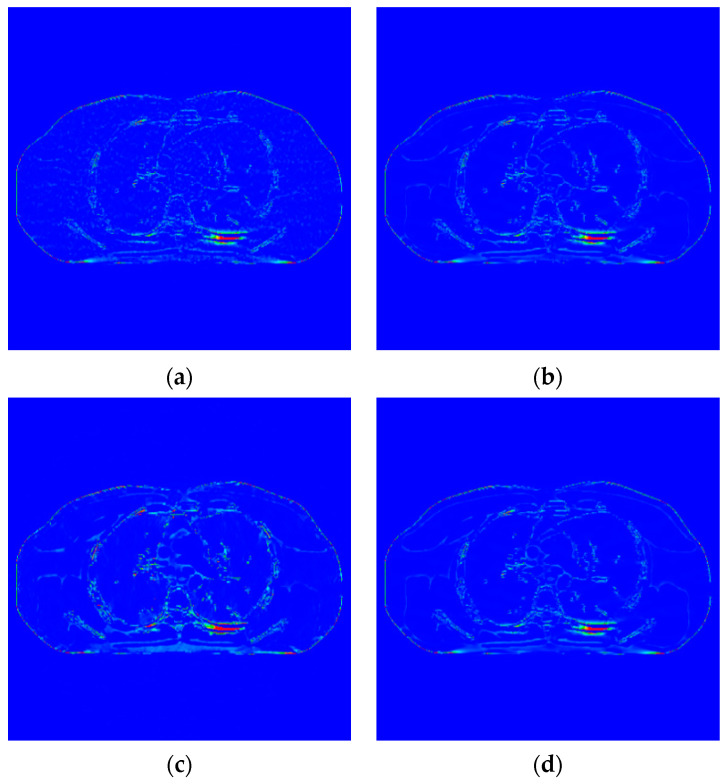
Image of difference of incident photon number $10^{5}$. (**a**) EP iterative reconstruction; (**b**) DCT image post-processing; (**c**) DL iterative reconstruction algorithm; (**d**) learning sparse transform.

**Figure 7:**
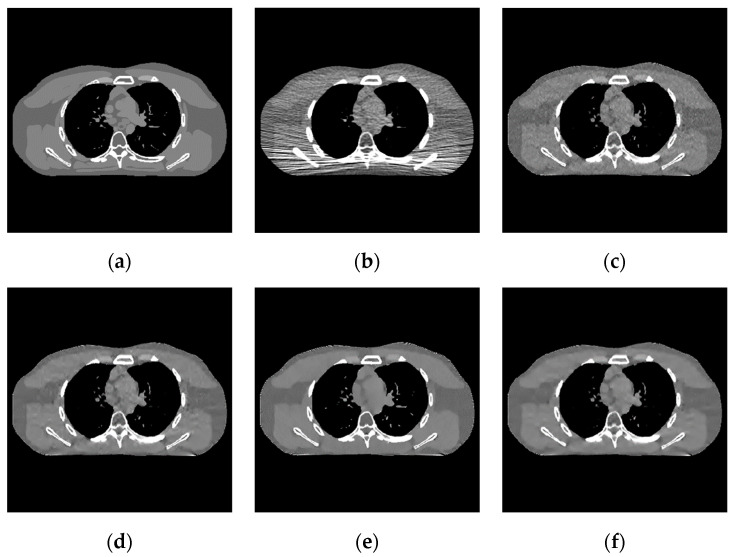
Image of the difference of incident photon number $10^{4}$. (**a**) real image; (**b**) FBP reconstruction; (**c**) EP iterative reconstruction; (**d**) DCT image post-processing; (**e**) DL iterative reconstruction; (**f**) learned sparse transformation.

After the training of sample data, for the test data, the number of incident photons $Q_{0}$ used in the experiment is $10^{5}$, $10^{4}$, and $5\times 10^{3}$ respectively. According to the corresponding relationship between the number of incident photons and the dose, the above three incident photon numbers are divided into three grades: Level 1—all most low-dose scanning; Level 2—standard low-dose scanning; Level 3—extremely low-dose scanning;

The proposed algorithm is compared with the FBP algorithm, EP iterative reconstruction algorithm, DCT based low-dose CT image post-processing algorithm, and dictionary-learning (DL) iterative reconstruction algorithm. The experimental results are shown from the global effect map, local organization enlarged image, and difference diagram, as shown in Figure 5, Figure 6 and Figure 7.

**Figure 8:**
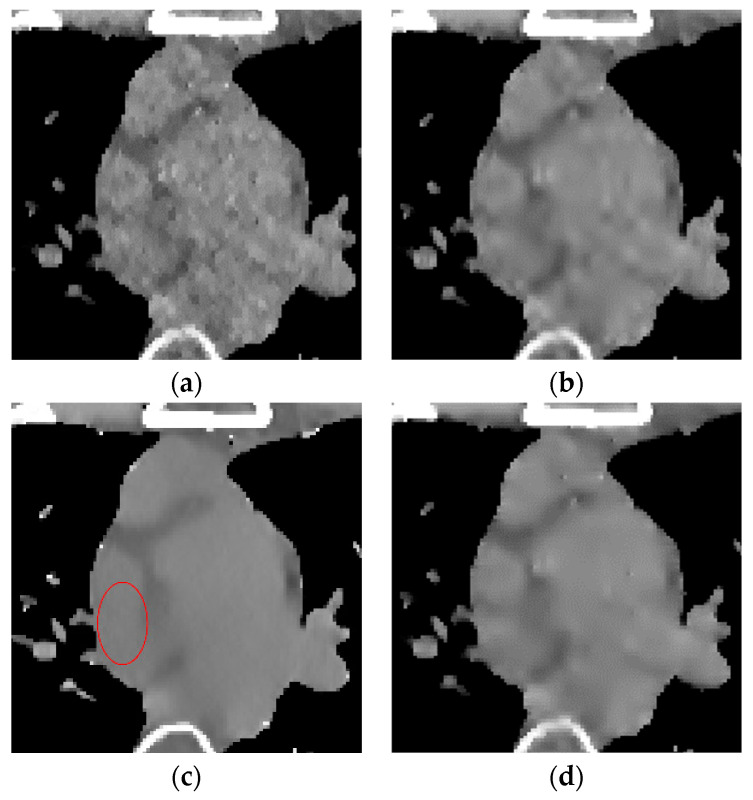
Enlarged local results of incident photon number $10^{4}$. (**a**) EP iterative reconstruction; (**b**) DCT image post-processing; (**c**) DL iterative reconstruction algorithm; (**d**) learning sparse transform.

**Figure 9:**
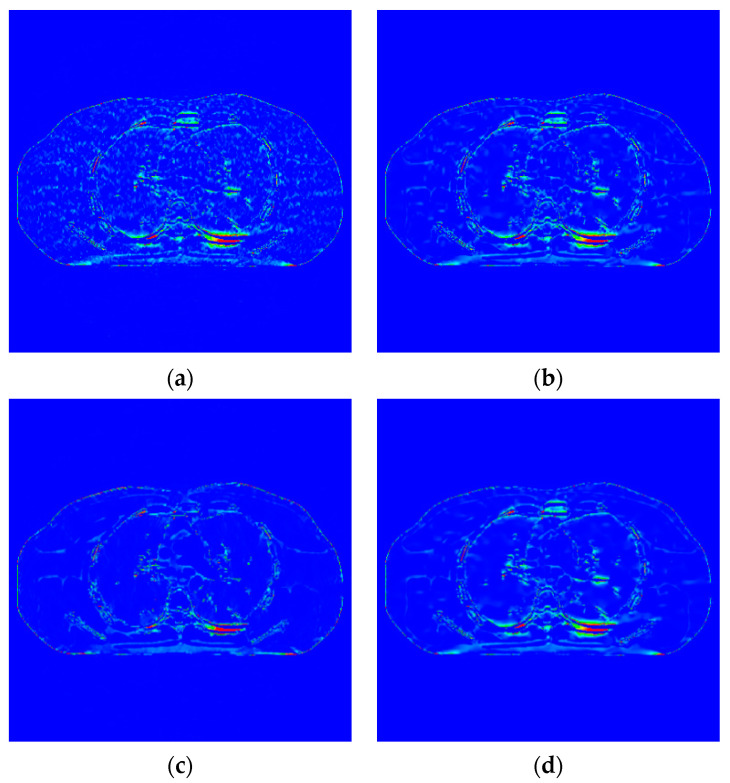
Image of difference of incident photon number $10^{4}$. (**a**) EP iterative reconstruction difference map; (**b**) DCT image post-processing; (**c**) DL iterative reconstruction algorithm; (**d**) learning sparse variation.

**Figure 10:**
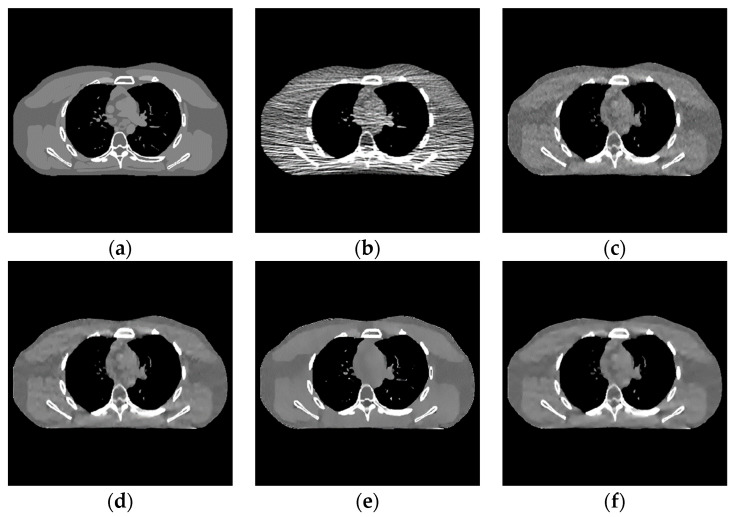
Experimental results of incident photon number $5\times 10^{3}$. (**a**) Real image; (**b**) FBP reconstruction; (**c**) EP iterative reconstruction image; (**d**) DCT image post-processing; (**e**) DL iterative reconstruction; (**f**) learning sparse transform.

The global effect map shows the overall effect of the experimental results; the local tissue enlarged image is used to observe the difference of the detailed information of the tissue structure. For the details that are difficult to be identified by the naked eye, the difference is shown by calculating the difference with the standard dose image, in which blue represents the minimum difference and red represents the maximum difference: the colder the color is, the closer it is to the standard measurement. The relatively complex tissue part in the middle of the CT image is selected for local magnification (Figure 8, Figure 9 and Figure 10) to facilitate each algorithm’s performance when the tissue structure is complex.

**Figure 11:**
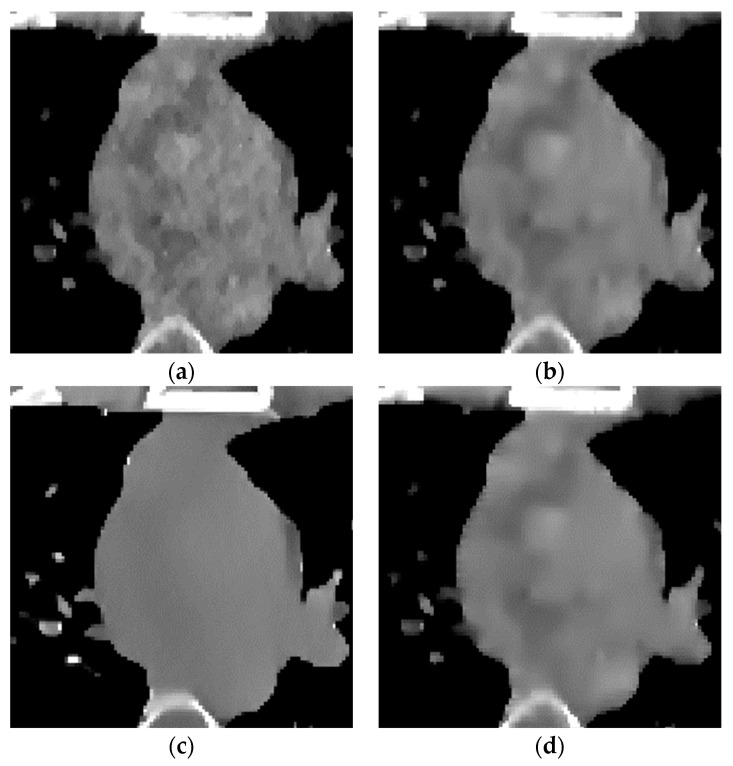
Enlarged local results of incident photon number $5\times 10^{3}$. (**a**) EP iterative reconstruction; (**b**) DCT image post-processing; (**c**) DL iterative reconstruction algorithm; (**d**) learning sparse transform.

**Figure 12:**
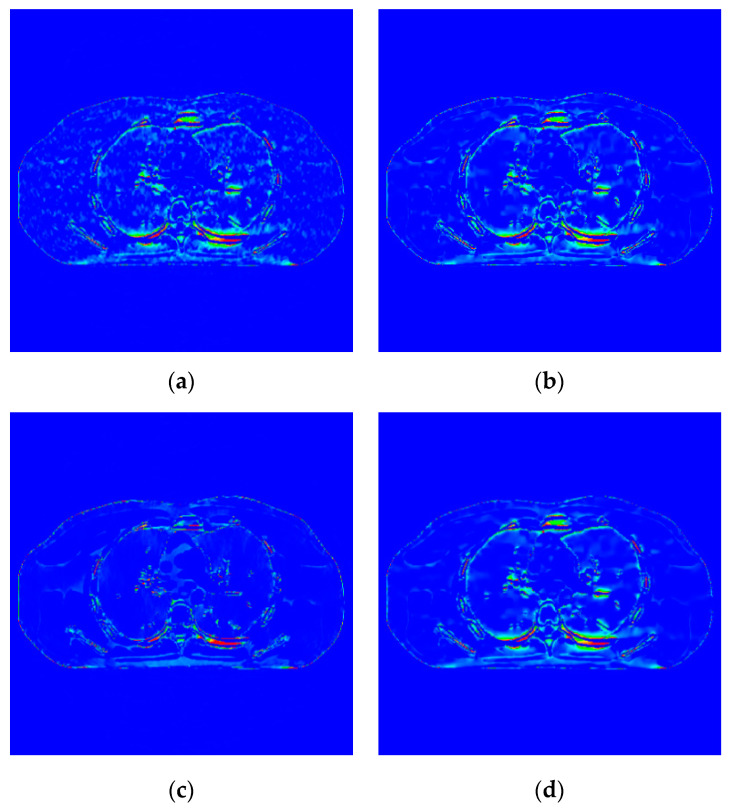
Difference image of incident photon number $5\times 10^{3}$. (**a**) EP iterative reconstruction difference image; (**b**) DCT image post-processing principle; (**c**) DL iterative reconstruction algorithm; (**d**) learning sparse transformation image.

The complex organizational structure algorithm performance is shown in Figure 11 and Figure 12.

### 3.3. Quantitative Index Analysis

**Figure 13:**
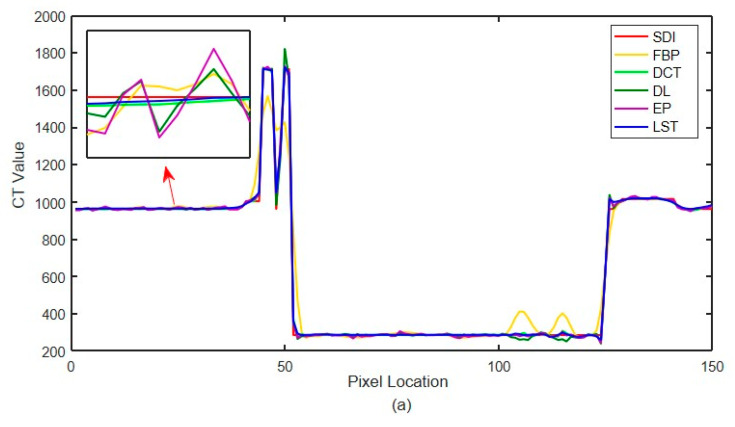

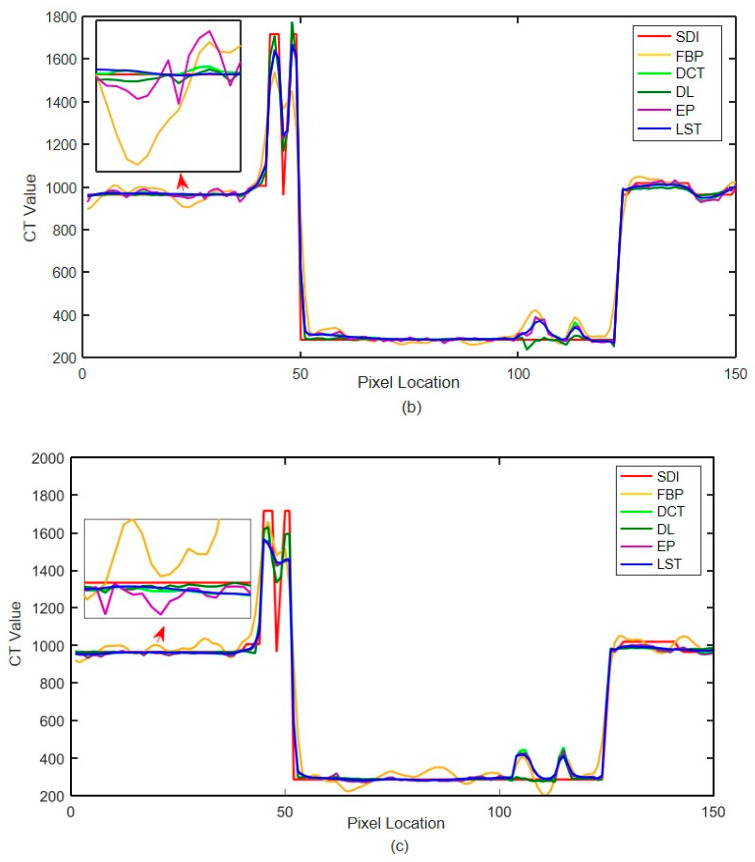
Local CT intensity curve. (**a**) The number of incident photons $10^{5}$; (**b**) the number of incident photons $10^{4}$; (**c**) the number of incident photons $5\times 10^{3}.$

The quantitative index evaluation criteria adopted in this paper are RMSE (HU) and (Structural Similarity) SSIM [46] to analyze the effect quantitatively. The values of three different incident photon numbers are shown in Table 1. Figure 13 shows the local CT value curve. The quality of each algorithm’s results is evaluated according to the proximity between the algorithm result curve and the standard dose image curve. In Figure 13, SDI is ‘Standard Dose Image’, FBP is ‘Filtered Back Projection’, LST is’ Learning sparse transformation’.

We use SI (MKS) or CGS as the primary unit in the experimental results. Imperial units can be used as secondary units (in parentheses). For example, one can write “15 Gb/cm^2^ (100 Gb/in^2^)”. An exception is when English units are used as identifiers in trade, such as “3½-in disk drive”. We avoid combining SI and CGS units, such as current in amperes and magnetic field in oersteds, as this often leads to confusion because equations do not balance dimensionally.

## 4. Discussion

1. When the number of incident photons is lower, there are some strip artifacts and speckle noises in the FBP algorithm (Figure 6b). A little speckle noise artifact is left in EP iterative reconstruction algorithm (Figure 6c), while low-dose CT image post-processing algorithms based on DCT (Figure 6d), DL dictionary iterative reconstruction algorithm (Figure 6e), and learning sparse transform image processing algorithm (Figure 6f) perform well in removing strip artifacts and noises. However, in a similar position of the organizational structure, the lower tissues of Figure 6c–f show a smooth transition. This smooth transition makes it difficult to distinguish the organizational structure.

Figure 7 shows the difference between the effect picture of each algorithm and the standard dose image. In Figure 7b–d, some light blue spots are evenly distributed in the region of interest. It corresponds to the realization of speckle noise in Figure 6c. However, in Figure 7c, more red areas appear at the edge of the organizational structure, indicating a large difference in the region. There are no large areas of blue spots and red areas in Figure 8b,d, and their color distribution is similar. Therefore, compared with the EP iterative reconstruction algorithm, the proposed algorithm can remove speckle noise in the low-dose scene and has no obvious advantage compared with the DCT low-dose CT image post-processing and the DL iterative reconstruction algorithm.

2. In the scene of low-dose scanning with the number of incident photons, Figure 8 shows that the image reconstructed by the classical FBP algorithm (Figure 8b) has a lot of uneven speckle-noise many strip artifacts. It makes the tissue nodes fuzzy and seriously interferes with the disease diagnosis. EP iterative reconstruction algorithm (Figure 8c) and low-dose CT image post-processing calculation based on DCT (Figure 8d), the DL iterative reconstruction algorithm (Figure 8e), and the learning sparse transform image post-processing algorithm (Figure 8f) are superior at eliminating strip artifacts. However, for speckle noise, other algorithms still have different degrees of noise retention after processing except for the DL iterative reconstruction algorithm.

The algorithm proposed in this paper (Figure 9d) retains the tissue’s edge information. It displays a better effect for removing speckle noise than EP iterative reconstruction algorithm (Figure 9a) and the DCT image post-processing algorithm (Figure 9b). The DL iterative reconstruction algorithm (Figure 9c) can effectively remove speckle noise. The point noise has a good removal effect, but the tissue edge is too smooth in the red circle. It makes the tissue boundary not clear enough. In the difference diagram (Figure 10), the light blue spots in the ROI of Figure 10a–d decrease in turn, and Figure 10c appears in the middle complex tissue structure. Light blue spots are consistent with the local enlarged image (Figure 9c).

3. In the scene with very low-dose scanning, Figure 11 shows that the image reconstructed by the FBP algorithm (Figure 11b) contains a lot of speckle noise and strip artifacts. Part of the organizational structure information is also indistinguishable between the EP iterative reconstruction algorithm (Figure 11c) and the low-dose CT image post-processing algorithm based on DCT (Figure 11d). The image post-processing algorithm (Figure 11f) also performs well for removing strip artifacts. However, the speckle noise still exists. The DL iterative reconstruction algorithm (Figure 11e) can remove the noise and artifacts well. However, the degree of over-smoothness will also increase.

The noise in Figure 12a,b,d is not removed completely. However, the edge information of organizational structure is retained. The edge information of organizational structure in Figure 12c is eliminated along with the noise. The color distribution of each sub-image in the difference diagram of Figure 13 is consistent with the visual observation.

Therefore, in the low-dose and very low-dose scenes, the learning sparse transform image post-processing algorithm proposed in this paper can achieve a better denoising effect than FBP algorithm, EP iterative reconstruction algorithm, and low-dose CT image post-processing algorithm based on DCT, and also requires less time than the DL iterative reconstruction algorithm, which needs training and does not need to obtain projection data.

To sum up, this paper conducted experiments with the learning sparse transform image post-processing algorithm in three different dose scenarios, as shown in Figure 8f, Figure 10f and Figure 13f. By comparing different groups of experiments, the experimental results show that the algorithm has a better denoising effect than EP iterative reconstruction and DCT image post-processing algorithm. Moreover, compared with the DL iterative reconstruction algorithm, the algorithm does not appear to over-smooth phenomena in the organization structure’s complex part. Moreover, it has a faster calculation speed than iterative calculation.

In the quantitative index analysis, we can make the following observation: by observing the two-table data, with the decrease of the dose, the RMSE value of each algorithm shows an increasing trend, which shows that the denoising ability of various algorithms is weakening with the increase of noise, and the SSIM index also decreases with the decrease of dose, indicating that the recovery ability of organization details of various algorithms is also poor. Simultaneously, the exponential performance of learning sparse transform is better than FBP, EP iterative reconstruction algorithm, and DCT image post-processing algorithm, but not as good as the DL iterative reconstruction algorithm learning prior information.

## 5. Conclusions

This paper proposes a low-dose CT image post-processing method based on learning sparse transform. The image post-processing method does not need to obtain the real projection data. The method reduces the research threshold, can realize offline processing, and is easy to use. Affected by image morphological component analysis, low-dose CT images can be decomposed into tissue structure and noise images. Then, the structure sparse representation matrix and noise sparse representation matrix are proposed to represent the two parts of the image and realize the purpose of denoising. In the experimental stage, phantom data were used as sample and experimental data. Each group’s experimental results were analyzed using visual effects and quantitative indicators under different incident photon numbers (representing different radiation doses). The proposed algorithm has better image quality from the visual effect than the traditional filter back-projection algorithm. Furthermore, it performs well in the quantitative index. Finally, the influence of parameter selection on image quality is also shown.

After adding a prior image information constraint, the method improves the expressive ability of prior information, and can reconstruct better image effects. In addition, this method avoids the dependence of classical prior image compressive sensing reconstruction and discriminative feature representation models on prior images, as well as the registration and matching problems of prior images and reconstructed images from different sources.

## Figures and Tables

**Table 1:** RMSE (HU) and SSIM of different algorithms.

	Incident Photon Umbers	FBP	EP Iterative Reconstruction	Post-Processing of DCT Image	DL Iterative Reconstruction Algorithm	Learning Sparse Transformation
RMSE	10^5^	59.3	26.4	26	25.7	25.8
	10^4^	74.1	39.5	38.4	33.5	38.3
	5 × 10^3^	88	49.3	49.2	39.8	44.3
SSIM	10^5^	0.82	0.95	0.985	0.984	0.983
	10^4^	0.545	0.891	0.94	0.966	0.955
	5 × 10^3^	0.472	0.884	0.927	0.958	0.937

## Data Availability

The data are available at https://github.com/xuehangzheng/PWLS-ULTRA-for-Low-Dose-3D-CT-Image-Reconstruction (accessed on 4 April 2022).
